# Transcriptional upregulation of human tissue kallikrein 6 in ovarian cancer: clinical and mechanistic aspects

**DOI:** 10.1038/sj.bjc.6603556

**Published:** 2007-01-23

**Authors:** S J C Shan, A Scorilas, D Katsaros, E P Diamandis

**Affiliations:** 1 Department of Laboratory Medicine and Pathobiology, University of Toronto, Toronto, Ontario, Canada; 2 Department of Pathology and Laboratory Medicine, Mount Sinai Hospital, 600 University Avenue, Toronto, Ontario, Canada M5G 1X5; 3 Department of Biochemistry and Molecular Biology, University of Athens, Athens, Greece; 4 Department of Obstetrics and Gynecology, Gynecologic Oncology and Breast Cancer Unit, University of Turin, Turin, Italy

**Keywords:** ovarian cancer, biomarker, serine protease, gene regulation, human kallikrein 6, human kallikreins

## Abstract

The human tissue kallikrein family (KLK for protein; *KLK* for gene) includes 15 members. Twelve kallikreins, including KLK6, are concurrently upregulated in ovarian cancer. However, the mechanism of this phenomenon remains unclear. In this study, we measured KLK6 expression in a large series of ovarian tissue cytosols and examined possible mechanisms of KLK6 up-regulation in ovarian cancer. Using a newly developed enzyme-linked immunosorbent assay (ELISA) with two monoclonal antibodies, we quantified KLK6 expression in ovarian tissue cytosols, and confirmed the upregulation of KLK6 in ovarian cancer and its unfavourable prognostic value. We then examined *KLK6* mRNA expression using reverse transcription–polymerase chain reaction and established its good concordance with KLK6 protein expression. This finding suggested that the *KLK6* gene is under transcriptional regulation. We then scrutinised a few mechanisms that could explain KLK6 upregulation. The relative abundance of two *KLK6* mRNA transcripts was studied; we found the same differential expression pattern in all samples, regardless of KLK6 levels. Genomic mutation screening of all exons and the 5′-flanking region of the *KLK6* gene identified two linked single-nucleotide polymorphisms in the 5′-untranslated region, but neither correlated with KLK6 expression. Ovarian cell lines were separately treated with five steroid hormones. None of the treatments produced significant effects on KLK6 expression. We conclude that KLK6 is transcriptionally upregulated in ovarian cancer, but probably not through alternative mRNA transcript expression, genomic mutation, or steroid hormone induction.

The human tissue kallikreins (KLKs) are a family of serine proteases that are aberrantly expressed in several cancer types. Most strikingly, 12 kallikreins (KLK2, KLK3, KLK4, KLK5, KLK6, KLK7, KLK8, KLK10, KLK11, KLK13, KLK14, and KLK15) are concurrently upregulated in ovarian cancer (Borgono and Diamandis, 2004). Many of these KLKs hold promise as prognostic and diagnostic biomarkers (Yousef *et al*, 2001, 2003b; Diamandis *et al*, 2002, 2003; Borgono *et al*, 2003; Kishi *et al*, 2003; Luo *et al*, 2003; Scorilas *et al*, 2004). Several of them have also been implicated in cancer progression and metastasis (Bernett *et al*, 2002; Magklara *et al*, 2003; Becker *et al*, 2004). Evidently, kallikrein upregulation in ovarian cancer has significant relevance to cancer detection, management, and treatment. Understanding the mechanisms underlying their upregulation will allow for a better definition of kallikreins' role in ovarian cancer pathophysiology and their clinical utility as prognostic and/or diagnostic biomarkers or therapeutic targets.

The human tissue kallikrein genes (*KLK*s) are located in tandem on chromosome 19q13.4, and show significant structural homology (Yousef and Diamandis, 2001). Hence, the 12 kallikreins concertedly overexpressed in ovarian cancer may share a common regulation machinery. On this basis, we investigated the regulation of one kallikrein, human tissue kallikrein 6, as a model system. The findings may also prove applicable to other kallikreins. The *KLK6* gene spans 10.5 kb of genomic sequence on the kallikrein locus (Figure 1A) (Yousef *et al*, 1999). The KLK6 protein is normally expressed in adult brain, salivary gland, lung, colon, kidney, breast, uterus, fallopian tube and placental tissues, and secreted into numerous biological fluids (Diamandis *et al*, 2000; Petraki *et al*, 2001). Ample evidence supports the overexpression/increase of human KLK6 in both ovarian tumours and presurgical serum of patients with ovarian cancer (Hoffman *et al*, 2002; Diamandis *et al*, 2003; Yousef *et al*, 2003a). Nevertheless, few experiments have been conducted to delineate the processes underlying this upregulation.

In this study, we first quantified KLK6 expression, using a newly developed immunoassay with two monoclonal antibodies, in a relatively large series of ovarian cancer tissue specimens, and confirmed the prognostic value of KLK6 for ovarian cancer. We then assessed the correlation between *KLK6* mRNA and KLK6 protein expression in order to determine whether KLK6 is under transcriptional or translational upregulation. Lastly, we examined the role of alternative transcripts, genetic aberrations, and steroid hormones in the upregulation of KLK6 in ovarian cancer.

The human tissue kallikreins exhibit considerable transcriptomic complexity. Through usage of alternative promoters and other mechanisms, each *KLK* gene can generate numerous alternative transcripts (Landry *et al*, 2003; Pampalakis *et al*, 2004). Some of these transcript variants have been shown to be specifically overexpressed in ovarian cancer (Dong *et al*, 2001, 2003; Magklara *et al*, 2001). *KLK6* has four alternative transcripts encoding for the full-length KLK6 protein and four that may encode for truncated proteins (Kurlender *et al*, 2005). The longest *KLK6* transcript (GenBank accession no. NM002774), also known as the ‘classical transcript’, consists of 1512 nucleotides and seven exons. *KLK6* ‘alternative transcript 1’ has 1517 nucleotides (GenBank accession no. AY318867); it lacks exon 1 but contains a unique sequence at the 5′-end of exon 2, denoted as exon 2A (Figure 1B). Christophi *et al* (2004) found that these two mRNA transcripts were expressed in a tissue-specific manner and were differentially regulated in response to central nervous system injury. Whether or not an analogous situation occurs in ovarian cancer has not been investigated. To this end, we differentially amplified these two transcripts and examined their expression patterns in ovarian tumour tissues.

Another potential regulatory mechanism of the kallikreins is genetic variation. Even though intragenic mutations have not been identified for the kallikrein genes examined to date, *KLK3* (Majumdar and Diamandis, 1999) and *KLK10* (Liu *et al*, 1996), a plethora of single-nucleotide polymorphisms (SNPs) exist within *KLK* coding and promoter/enhancer regions and may have clinical significance. For example, SNPs in the *KLK3* promoter may account for individual variation in serum prostate-specific antigen levels and even cancer susceptibility (Tsuyuki *et al*, 1997; Xue *et al*, 2000; Cramer *et al*, 2003). Similarly, a nonsynonymous SNP in exon 3 of *KLK10* is associated with a higher prostate cancer risk (Bharaj *et al*, 2002). The *KLK6* locus in ovarian cancer patients has not previously been scrutinised for genetic aberrations. Hence, we sequenced all *KLK6* exons and the 5′-flanking region for ovarian tumours with various KLK6 levels to determine if genetic aberrations may account for the upregulation of KLK6 in ovarian cancer.

Lastly, various *in vitro* and *in vivo* studies collectively demonstrate that most, if not all, kallikreins are under steroid hormone regulation in endocrine-related tissues and cell lines (Borgono and Diamandis, 2004). Whereas some kallikreins, such as *KLK3* (Riegman *et al*, 1991) and *KLK4* (Nelson *et al*, 1999), are regulated by androgens, others, such as *KLK5* (Yousef and Diamandis, 1999), are more responsive to oestrogens. A role for the steroid hormones in the regulation of *KLK6* was first suggested by the discovery of several hormone-related response elements, including sterol regulatory element binding protein 1 and 2, progesterone receptor binding site and cAMP response element-binding protein in the 5′-flanking region of the *KLK6* gene (Christophi *et al*, 2004). It was subsequently supported by the findings that KLK6 was significantly up-regulated by norgestrel, oestradiol and DHT in the breast cancer cell line BT474 (Yousef *et al*, 1999). In this study, we examined whether or not steroid hormones regulated KLK6 expression in ovarian cancer cell lines.

Our results confirmed the value of KLK6 as an unfavorable prognostic biomarker for ovarian cancer. We also found KLK6 to be under transcriptional regulation. Our data have further suggested a minor role for alternative transcripts, genetic mutations and steroid hormones in the regulation of human kallikrein 6 in ovarian cancer.

## MATERIALS AND METHODS

### Tissue sample collection

We examined 259 patients with ovarian cancer, 49 with benign ovarian conditions (including endometriosis, mucinous cystadenomas, serous cystadenomas, dermoid cysts, ovarian benign teratomas and corpus luteum), 43 with nonovarian tumours that metastasised to the ovary, and 34 apparently normal women. Participants' age ranged from 19 to 89 years, with a median of 58 years. All tissue samples were collected between April 1988 and April 2003 at the Gynecologic Oncology and Breast Cancer Unit, University of Turin, Italy. Identical specimen collection and processing protocols were used for all participants. During surgery, histologic examination was performed on the ovarian tissues through intrasurgery frozen section analysis, which allowed representative portions of each tumour containing more than 80% tumour cells to be selected. All tumour samples collected were snap-frozen in liquid nitrogen and stored at −80°C until extraction.

At the time of surgery, clinicopathologic information including tumour stage, grade, histotype, residual tumour size, and debulking success were documented. Tumours were staged according to the Fédération Internationale des Gynaecologistes et Obstetristes (FIGO) criteria (Pettersson, 1994) and graded according to the Day *et al*, (1975) protocol. Tumour histotypes were classified based on the World Health Organization (WHO) and FIGO recommendations (Serov *et al*, 1973). Patients with ovarian cancer at all stages (I–IV) and grades (1–3) were represented in our study. Of the 259 ovarian tumours, the majority (110; 42%) were of serous papillary histotype, followed by endometrioid (46; 18%), undifferentiated (33; 13%), mucinous (20; 8%), clear cell (18; 7%), or other nonepithelial types (24; 9%).

After surgery, we monitored the patients with ovarian cancer for clinical response to chemotherapy and survival outcomes for a median duration of 52 months. Follow-up information was available for 232 patients, of whom 147 (63%) had relapsed and 117 (50%) had died.

All investigations were carried out according to the ethical standards of the Helsiniki Declaration of 1975 (revised in 1983), and were approved by the Institute of Obstetrics and Gynecology, Turin, Italy, and the Institutional Review Board of Mount Sinai Hospital, Toronto, Canada.

### Preparation of cytosolic extracts

Snap-frozen tissues (20–100 mg) were homogenised in liquid nitrogen to a fine powder and added to 10 volumes of extraction buffer (50 mM Tris, pH 8.0, 150 mM NaCl, 5 mM EDTA, and 1% NP-40 surfactant). The resulting mixtures were incubated on ice for 30 min, with repeated vortexing every 10 min, and then centrifuged at 14 000 r.p.m at 4°C for 30 min. The supernatant (cytosolic extract) was collected and stored at −80°C until further experiments. Total protein concentration of the extracts was determined using the bicinchoninic acid method. Bovine serum albumin was used as standard (Pierce Chemical Co., Rockford, IL, USA).

### Measurement of KLK6 and CA125 protein expression in ovarian cytosolic extracts

The concentration of KLK6 was quantified with a highly sensitive and specific ‘sandwich-type’ immunoassay recently developed in our laboratory. Two KLK6-specific monoclonal mouse antibodies were used: the coating antibody (clone 27-4) and the detection antibody (clone E24). The assay has a detection limit of 0.05 *μ*g l^−1^, a dynamic range up to 20 *μ*g l^−1^, and does not cross-react with any other members of the kallikrein family. White polystyrene microtiter plates were first coated with 100 *μ*l of the coating antibody solution (5 mg l^−1^ of the anti-KLK6 monoclonal antibody clone 27-4 in 50 mM Tris-HCl buffer, 0.05% sodium azide, pH 7.8). The plates were incubated overnight at room temperature and then washed three times with the washing buffer (5 mM Tris-HCl buffer, 150 mM NaCl, 0.05% Tween-20, pH 7.8). After washing, assay standards (KLK6 recombinant protein produced in-house (Diamandis *et al*, 2000)) or ovarian cytosolic extracts were added to the wells in duplicates (100 *μ*l well^−1^) after being diluted two-fold in assay buffer (50 mM Tris-HCl buffer, 6% bovine serum albumin, 0.01% goat globulin, 0.005% mouse globulin, 0.1% bovine globulin, 0.5 M KCl, 0.05% sodium azide, pH 7.8). The plates were incubated for 2 h with shaking at room temperature and washed six times. Subsequently, 100 *μ*l of the biotinylated detection antibody solution (250 *μ*g l^−1^ anti-KLK6 monoclonal antibody clone E24 in assay buffer) was added to each well. The plates were incubated for 1 h at room temperature with shaking and washed six times. Then, 100 *μ*l of alkaline phosphatase-conjugated (ALP) streptavidin solution (Jackson ImmunoResearch) diluted 20 000-fold in bovine serum albumin buffer (6% bovine serum albumin, 50 mM Tris-HCl buffer, 0.05% sodium azide, pH 7.8) was added to each well. The plates were incubated for 15 min with shaking at room temperature and washed six times. Lastly, 100 *μ*l of substrate buffer (0.1 M Tris-HCl buffer, 0.1 M NaCl, 1 mM MgCl_2_, pH 9.1), containing 1 mM diflunisal phosphate (DFP), was added to each well and incubated for 10 min with shaking at room temperature. Immediately after incubation, 100 *μ*l of developing solution (1 M Tris-HCl buffer, 0.4 M NaOH, 2 mM TbCl_3_, 3 mM EDTA) was added to each well and incubated for 1 min with shaking at room temperature. The fluorescence was measured with a time-resolved fluorometer, the Cyberfluor 615 Immunoanalyzer (MDS Nordion), as described elsewhere (Christopoulos and Diamandis, 1992). The KLK6 concentrations in ng ml^−1^ were converted to ng of KLK6 mg^−1^ of total protein to adjust for the amount of tumour tissue extracted.

CA125 levels (KU mg^−1^) of ovarian tissue samples were measured with the Immulite 2000 assay (Diagnostic Products Corporation, Los Angeles, CA, USA).

### Statistical analysis

The distribution of KLK6 concentration in the ovarian tumour cytosols was non-Gaussian. Therefore, we used the nonparametric Mann–Whitney *U*-test to determine differences among the four groups of samples. This test treated KLK6 concentration in the tumour cytosols (ng mg^−1^ of total protein) as a continuous variable. We also assessed the association between KLK6 and CA125 levels by determining the Spearman rank correlation coefficient (*r*) and associated *P*-values. The ovarian cancer patients were classified as KLK6-positive or KLK6-negative using the median (2.83 ng mg^−1^) as the cutoff point. The relationship between KLK6 expression and several clinicopathologic variables was analysed with the *χ*^2^ test and the Fisher's exact test, as appropriate.

We then assessed the impact of KLK6 on patient survival by calculating the hazard ratio (HR), which is the relative risk of relapse or death in the KLK6-positive group, using the Cox univariate and multivariate proportional hazard regression models (Cox, 1972). Whereas progression-free survival (PFS) was defined as the time interval between the first surgery and the identification of recurrence or metastatic disease, overall survival (OS) was defined as the time interval between the first surgery and death. The multivariate models were adjusted for KLK6 expression in tumours and other clinicopathologic variables that may affect survival, including age, tumour grade, CA125 level, and histotype. We only included patients for whom the status of all variables was known in the multivariate models. Lastly, we constructed Kaplan and Meier (1958) PFS and OS curves for the KLK6-positive and KLK6-negative patients. The differences between the survival curves were assessed for statistical significance using the log rank test (Mantel, 1966). Patients were also classified as CA125-positive and CA125-negative using the median 1330 U mg^−1^ as cutoff, and we constructed Kaplan–Meier PFS and OS curves to evaluate the prognostic significance of CA125.

### RNA extraction and RT–PCR

We selected eight tumour tissues (H1–H8) that over-expressed KLK6 (mean: 12.44 ng mg^−1^), eight samples (L1–L8) that expressed minimal KLK6 (mean: 0.06 ng mg^−1^), four benign ovarian samples (B1–B4; mean KLK6 expression: 0.05 ng mg^−1^), and one normal ovarian sample (N1; KLK6 expression: 0.02 ng mg^−1^). Total RNA was isolated from these samples using the TRIzol® method (Life Technologies Inc., Gaithersburg, MD, USA) as directed by the manufacturer. The yield and purity were determined spectrophotometrically by measuring the absorbance of aliquots at 260 and 280 nm. Two micrograms of total RNA were then reverse transcribed using the SuperScript™ preamplification system (Life Technologies Inc.) in a final volume of 20 *μ*l. As control for the reverse transcription–polymerase chain reaction (RT–PCR), we used commercially available human brain total RNA (Clonetech Inc., Mountain View, CA, USA). To test the success of our reverse transcription, 1 *μ*l of each cDNA product was PCR-amplified using primers specific for the housekeeping gene, *β*-actin (for primer sequences see Table 1), and visualised on 1.8% agarose gels stained with ethidium bromide.

### PCR amplifications

**Figure 1:**
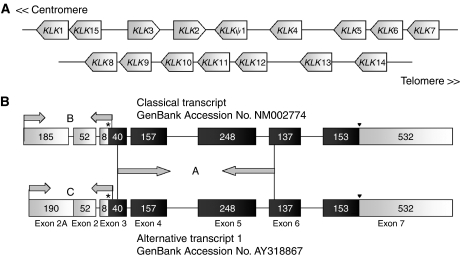
(**A**) Fifteen *KLK* genes are located in tandem on chromosome 19q13.4. (**B**) Genomic structures of the *KLK6* ‘classical transcript’ (GenBank accession no. NM002774) and the ‘alternative transcript 1’ (GenBank accession no. AY318867). Black regions denote coding exons, white regions denote 5′- and 3′-untranslated regions. Segment A arrows indicate primer locations for PCR amplification of all full-length *KLK6* transcripts. Segments B and C arrows indicate primer locations for differential PCR amplification of the *KLK6* ‘classical transcript’ and the ‘alternative transcript 1’, respectively. Asterisks and triangles indicate translation start sites and stop codons, respectively.

To amplify the mRNA transcript variants that encode the full-length KLK6 protein (GenBank accession no. NM002774, AY318867, AY318869, and BC015525), we designed primers (for sequences see Table 1; Figure 1B) that anneal to a common region for all four variants (nucleotides 257–709; numbers refer to GenBank accession no. AY318867).

One microliter of first-strand cDNA template was amplified in a 25 *μ*l reaction mixture containing 10 × PCR buffer (Qiagen Inc., Mississauga, ON, USA), 100 *μ*M dNTPs (deoxynucleoside triphosphates), 2.5 U of HotStar *Taq* DNA polymerase (Qiagen Inc.) and 100 ng of the appropriate primers in an Eppendorf thermocycler. Cycling conditions were as follows: enzyme activation at 95°C for 15 min; 32 cycles of denaturation at 95°C for 30 s, annealing at 65°C for 30 s, and extension at 72°C for 30 s; followed by a final extension at 72°C for 10 min. The *KLK6* PCR product was visualised on 1.8% agarose gels stained with ethidium bromide. Gels were photographed under UV light with the Speedlight Gel Documentation System (Lightools Research, Encinitas, CA, USA). Two independent observers examined the images to determine the presence or absence of *KLK6* PCR-generated bands. Each amplification experiment was performed twice to ensure reproducibility of data.

To differentially amplify the ‘classical transcript’ and ‘alternative transcript 1’ (GenBank accession no. NM002774 and AY318867, respectively), which differ only in their 5′-untranslated regions (UTRs), we used two unique forward primers and the same reverse primer (for sequences, see Table 1; Figure 1B). The PCR reactions amplified nucleotides 1–285 for the ‘classical transcript’ (numbers refer to the sequence NM002774), and nucleotides 1–290 for ‘alternative transcript 1’ (numbers refer to the sequence AY318867). We ensured that the two primer sets had comparable annealing efficiency by conducting parallel PCR reactions with each primer set and equal amounts of plasmid DNA. These reactions generated PCR bands of similar intensity (Figure 5B). Cycling conditions for both amplifications were the same as described above, except for the annealing temperature, which was 61°C for both reactions. Only tumour samples H1–H8, which expressed high kallikrein 6 protein and mRNA, were used in this experiment. Normal human brain and ovarian cDNA templates were used as controls.

### Genomic DNA extraction and mutation screening

An even representation of ovarian tumour tissues expressing high KLK6 (*n*=9; mean=11.72 ng mg^−1^) and low KLK6 (*n*=9; mean=0.09 ng mg^−1^), benign ovarian tissues (*n*=8; mean: 0.09 ng mg^−1^), non-ovarian tumours that metastasised to the ovary (*n*=6; mean: 0.04 ng mg^−1^), and normal ovarian tissues (*n*=13; mean: 0.06 ng mg^−1^) were selected for genomic DNA extraction and sequencing. DNA was extracted using the QIAamp DNA mini Kit (Qiagen Inc.) according to the manufacturer's protocols, and its yield and purity were determined spectrophotometrically by measuring the absorbance of aliquots at 260 and 280 nm.

To design primers for mutation screening, we used *KLK6* ‘alternative transcript 1’ as template, as it was found to be the dominant transcript in the ovaries. We designed seven sets of primers amplifying all exons and their flanking intronic sequences, as well as the 5′-flanking region (601 bp upstream of the putative transcription start site, numbers refer to the *KLK6* genomic sequence, GenBank accession no. AF149289). Sequences of all primers are shown in Table 1. Polymerase chain reaction was carried out with 2 *μ*g of genomic DNA in a 25 *μ*l reaction mixture containing 10 × reaction buffer, 100 *μ*M of dNTP, 1 U of *Pfu* Turbo Polymerase (Stratagene, La Jolla, CA, USA), and 100 ng of the appropriate primers. *Pfu* Turbo DNA polymerase was employed because of its high fidelity. The PCR cycling conditions were as follows: 3 min of enzyme activation at 95°C, 35 cycles of 1 min denaturation at 95°C, 45 s of annealing at a primer-dependent temperature (Table 1), and 1 min of extension at 72°C, followed by 10 min of final extension at 72°C. The extension time for each cycle was prolonged to 1 min and 30 s when amplifying the 5′-flanking region and exon 7, to compensate for the length of these PCR products. The success of the PCR reactions were evaluated by running 5 *μ*l of each PCR product on 1.8% agarose gels stained with ethidium bromide. The remaining of the PCR products was purified by treatment with exonuclease I (0.25 U) and shrimp alkaline phosphatase (0.25 U) (USB, Cleveland, OH, USA). Mixtures were incubated at 37°C for 15 min, and then at 85°C for 15 min. After purification, PCR products were directly sequenced using an automated DNA sequencer. The PCR product was sequenced again in the opposite direction if sequencing results showed a departure from the published sequence in GenBank (accession no. AF149289).

### Cell lines and hormonal stimulation experiments

BG-1, CaOV3, and OVCAR-3 are ovarian cancer cell lines, purchased from the American Type Culture Collection (ATCC, Rockville, MD, USA) and grown in RPMI media (Invitrogen, Carlsbad, CA, USA) containing 10% fetal bovine serum (FBS) (HyClone Laboratories, Inc., Logan, UT, USA). The human ovarian surface epithelium (hOSE) cell line was obtained from Dr Nelly Auersperg at the University of British Columbia. It was originally established by scraping human ovarian surface epithelial cells (*n*=3) from the ovarian surface of overtly normal ovaries during laparoscopies for nonmalignant disorders (Kruk and Auersperg, 1992). The hOSE cells were cultured in medium 199 : MCDB105 (1 : 1) (Life Technologies Inc.) with 10% FBS (HyClone).

All cell lines were cultured in plastic flasks to near confluency, aliquoted into six-well tissue culture plates, and then cultured to 75% confluency. Twenty-four hours before the hormonal stimulation treatments, the culture medium was changed into each cell line's respective medium containing 10% charcoal-stripped fetal bovine serum. Cells were grown in the new media for 24 h.

The steroid hormones used were estroadiol, aldosterone, dihydrotestosterone (DHT), dexamethasone, and norgestrel. Each steroid hormone was dissolved in 100% ethanol, and added to the culture media in each well at a final concentration of 10^−8^ M and less than 1% ethanol. Controls were the cells stimulated with an equal amount of ethanol only. All cells were grown for 7 days after stimulation and the cell culture supernatant was collected for KLK6 measurement using the aforementioned immunoassay. These experiments were repeated three times to ensure reproducibility of results.

## RESULTS

### Distribution of KLK6 concentration in ovarian tissues

**Figure 2:**
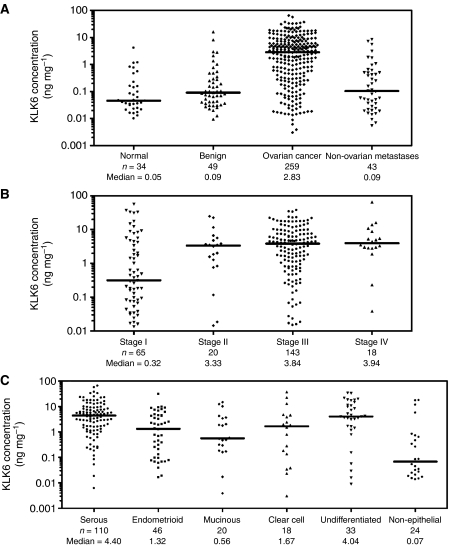
Distribution of KLK6 concentration in ovarian tissue cytosols. (**A**) Distribution of KLK6 concentration in apparently healthy women (normal); women with benign ovarian conditions (benign); women with ovarian cancer; and women with nonovarian cancers that metastasised to the ovaries (nonovarian metastases). (**B**) Distribution of KLK6 concentration in women with different stages of ovarian cancer. (**C**) Distribution of KLK6 concentration in various histotypes of ovarian cancer. Horizontal lines represent medians. *n*, number of samples.

The KLK6 protein concentration in normal ovarian tissues ranged from 0.01 to 4.11 ng mg^−1^ of total protein, with a median of 0.05. Both benign ovarian tissues (median: 0.09; range: 0.01–16 ng mg^−1^) and nonovarian tumours that metastasised to the ovary (median: 0.09; range: 0.01–8.56 ng mg^−1^) also had relatively low KLK6 expression. Conversely, the protein concentration of KLK6 in the 259 ovarian tumour cytosols ranged from 0.01 to 65 ng mg^−1^ of total protein, with a median of 2.83 (Table 2; Figure 2A). We performed the nonparametric Mann-Whitney *U*-test to analyse the differences in KLK6 expression among the four groups of ovarian tissue samples. KLK6 expression in ovarian tumour cytosols was found to be 57-fold higher than normal and 31-fold higher than benign and nonovarian metastatic tumours (*P*<0.0001). We used the median as the cutoff value to categorise the ovarian tumours as KLK6-positive or KLK6-negative.

### Relationships between KLK6 status and other clinicopathologic variables

**Figure 3:**
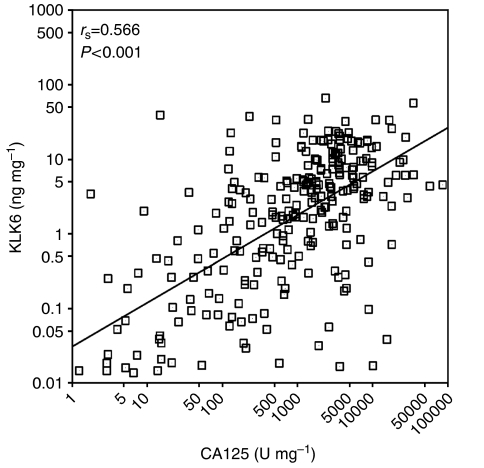
Correlation between tissue CA125 and KLK6 levels. *r*_s_, Spearman's correlation coefficient.

We categorised KLK6-positive and KLK6-negative patients according to clinicopathologic variables including tumour stage, grade, histotype, debulking success and response to chemotherapy (Table 3). The strength of the relationships between KLK6 and these variables was evaluated with either the *χ*^2^ test or the Fisher's exact test, as appropriate. We found that patients with KLK6-positive tumours more frequently had late stage (stage III/IV) disease (*P*<0.001) (Figure 2B), higher tumour grades (*P*=0.04), suboptimal debulking (*P*=0.002), and serous and undifferentiated histotypes (*P*<0.001) (Figure 2C); but KLK6 expression had no relationship with response to chemotherapy. A weak positive correlation between tissue CA125 and KLK6 expression in ovarian cancer (Spearman correlation *r*_s_=0.566; *P*<0.001) was also observed (Figure 3).

### Univariate and multivariate survival analysis

**Figure 4:**
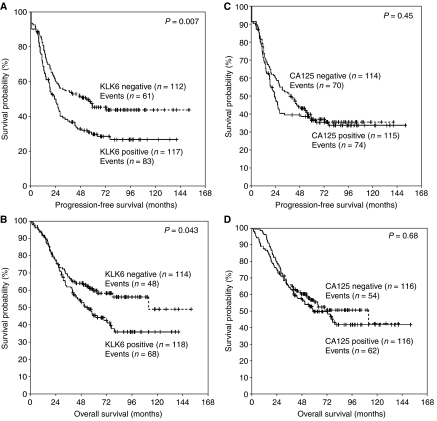
Kaplan–Meier survival curves for progression-free survival (**A**) and overall survival (**B**) in patients with KLK6-positive and KLK6-negative ovarian tumours. *n*, number of samples. Kaplan–Meier survival curves for progression-free survival (**C**) and overall survival (**D**) in patients with CA125-positive and CA125-negative ovarian tumours. *n*, number of samples.

The association between KLK6 protein expression and patient survival is summarised in Table 4. Univariate Cox regression analysis showed that KLK6-positive patients were at an increased risk of relapse (HR=1.56; *P*=0.008) and death (HR=1.46; *P*=0.045) in comparison with KLK6-negative patients. When KLK6 expression was treated as a continuous variable (after logarithmic transformation of data), its correlation with patient survival remained significant (HR=1.45 for PFS, *P*<0.001; and 1.37 for OS, *P*=0.004). Other variables such as histological type and grade, but not age, had even higher HRs for both PFS and OS. In multivariate Cox regression analysis, the relationship between KLK6 status and survival outcome was no longer significant. The same was true for patient age. Tumour grade remained a significant predictor of both PFS and OS, though histological type was no longer associated with OS. Kaplan–Meier survival curves showed significant association between KLK6 status and both PFS and OS (Figure 4A and B). On the contrary, Kaplan–Meier survival curves showed that CA125 did not bear prognostic significance (Figure 4C and D).

### Expression of *KLK6* mRNA transcripts in ovarian tumours

**Figure 5:**
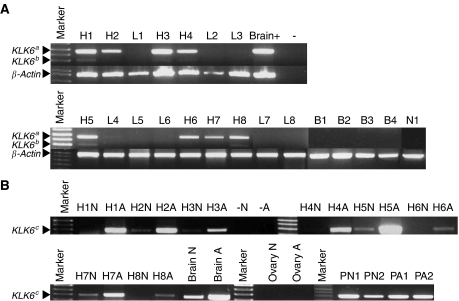
(**A**) *KLK6* mRNA expression in ovarian tumours with high KLK6 protein (H1–H8) and low KLK6 protein (L1–L8), four benign ovarian samples (B1–B4), one normal ovarian sample (N1), and one normal brain sample. *KLK6*^*a*^: all *KLK6* full-length mRNA transcripts. *KLK6*^*b*^: *KLK6* splice variant missing exon 4 (GenBank accession no. AY279383). *β*-Actin was used as control for the quality of cDNA. Note the expression of mRNA only in tumours with high KLK6 protein. (**B**) Differential expression patterns of KLK6 ‘classical transcript’ and ‘alternative transcript 1’ in eight high KLK6 protein expressing tumours (H1–H8). N: amplification using primers for the ‘classical transcript’; A: amplification using primers for the ‘alternative transcript 1’. KLK6^c^ denotes both ‘A’ and ‘N’ amplicons, which are 285 and 290 bp, respectively. Brain and ovarian normal cDNA were used as controls. Note predominance of ‘alternative transcript 1’ expression in all cases, as well as in the brain tissue (control). PN1, PN2 were two samples of plasmid DNA containing ‘N’ amplicon as insert, reamplified using ‘N’ primers. PA1, PA2 were two samples of plasmid DNA containing ‘A’ amplicon as insert, reamplified using ‘A’ primers. Note that when starting material was the same, the two sets of primers generated PCR bands of similar intensity.

The collective expression of all *KLK6* mRNA transcripts encoding the full-length protein was assessed in 16 ovarian tumour samples, eight with KLK6 overexpression (H1–H8) and eight with minimal KLK6 expression (L1–L8). Whereas *KLK6* mRNA expression was significant in H1–H8, it was undetectable in L1–L8 (Figure 5A). For some of the high-KLK6 expressing samples, we observed an additional, shorter, and much weaker band generated by PCR amplification. After sequencing, we confirmed this band to represent a previously identified splice variant (GenBank accession no. AY279383), which lacks exon 4 (Kurlender *et al*, 2005). Four benign and one normal ovarian tissue samples expressing low KLK6 were used as controls. All had undetectable *KLK6* mRNA expression (Figure 5A).

We also examined the relative expression of two most abundant *KLK6* mRNA transcripts. For each of tumour samples H1–H8, we assessed the differential expression of the ‘classical transcript’ and ‘alternative transcript 1’. Normal brain and ovarian cDNA templates were used as controls. For all samples studied, excluding normal ovarian tissues which showed no expression for either transcript, ‘alternative transcript 1’ was by far the dominant transcript (GenBank accession no. AY317768) (Figure 5B).

### SNPs in the 5′-UTR of *KLK6* ‘alternative transcript 1’

As ‘alternative transcript 1’ was found to be the more abundant transcript (Figure 5B), we used it as the template for our mutation screening experiments. We amplified and sequenced all *KLK6* exons and the 5′-flanking region. No mutations were found in any of the coding exons of *KLK6*. However, we identified two previously unpublished single nucleotide polymorphisms (SNPs) located in a (GT)_4_ microsatellite in the 5′-untranslated region (exon 2A). The SNPs are located at 55 and 60 bp downstream of the putative transcription start site (Pampalakis *et al*, 2004), and 195 and 190 bp upstream of the start codon, respectively. G was the more frequent allele for both SNPs. The two SNPs were in linkage disequilibrium (the dependence of an allele at one locus on alleles at another locus), as the T allele was found at the second SNP only if the T allele was also present at the first SNP. We observed six different haplotypes across all samples studied. None of the haplotypes significantly correlated with KLK6 expression or sample type.

According to the published GenBank sequence AY318876, the microsatellite in this region consists of four GT repeats (GT)_4_. However, according to our findings, the most prevalent haplotype (51%) has a (GT)_3_ microsatellite owing to a single nucleotide change from T to G at basepair 55. As all tumour samples were collected from Italian subjects, we examined if the difference in the microsatellite length was due to race. We sequenced three normal ovarian tissues obtained from Mount Sinai Hospital, Ontario, Canada, and found them to also have Haplotype B.

### Hormonal regulation of KLK6 in ovarian cancer

**Figure 6:**
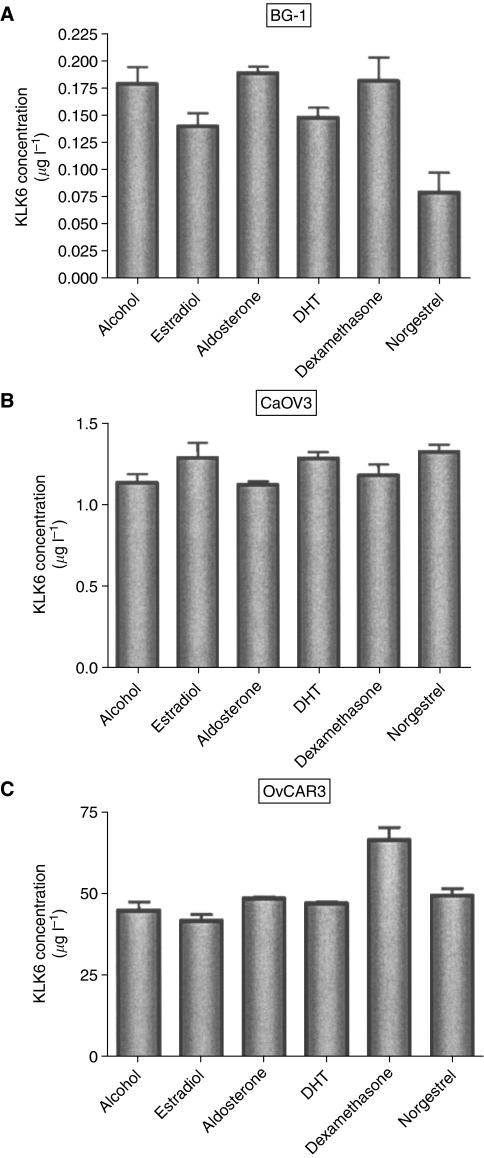
Ovarian cancer cell lines BG-1, CaOV3, OvCAR3 were stimulated with 10^−8^ M of estradiol, aldosterone, DHT, dexamethasone, norgestrel, or alcohol (control). KLK6 protein concentration in supernatants was quantified by immunoassay. No major changes were seen with any steroid hormone stimulation. Error bars represent standard errors of the mean.

We examined the effects of steroid hormones on KLK6 expression in BG-1, CaOV3 and OVCAR-3 epithelial ovarian cancer cell lines, as well as the hOSE cell line. As illustrated in Figure 6, none of the steroid hormones produced any significant changes in KLK6 expression for the ovarian cancer cell lines. The hOSE cell line did not produce detectable KLK6 under any treatment conditions (data not shown).

## DISCUSSION

Ovarian cancer is the most lethal gynaecologic malignancy in developed countries (Mor *et al*, 2005). The high morality rate of this disease could be attributed to difficulties underlying its early detection, the disease's intrinsic aggressiveness, and our meagre understanding of its aetiology. The development of novel diagnostic and prognostic biomarkers will facilitate early detection and more individualised and efficient treatment plans for ovarian cancer patients. Of the plethora of novel potential biomarkers identified in recent years, those that also have clinical relevance to cancer initiation and progression are the most valuable, as they may serve the dual role as biomarkers as well as therapeutic targets.

Serine proteases are among these newly identified tumour-associated biomarkers (Duffy, 1996). Accumulating evidence suggests that the human tissue kallikreins, a family of secreted serine proteases ubiquitously expressed in human tissues and biological fluids, have prognostic and diagnostic value in various cancer types (Borgono and Diamandis, 2004). Some KLKs have also been shown to play a role in extracellular matrix degradation, which in turn facilitates tumour invasion and metastasis (Borgono *et al*, 2004). Human kallikrein 6, in particular, is elevated in presurgical serum of ovarian cancer patients (Diamandis *et al*, 2003), as well as overexpressed in ovarian tumour tissues (Hoffman *et al*, 2002), and bears prognostic and diagnostic significance. KLK6 is a trypsin-like enzyme that can degade *in vitro* laminin and fibronectin (Bernett *et al*, 2002), as well as other basic constituents of the extracellular matrix (ECM) and the basement membrane (Magklara *et al*, 2003), and could be linked to tumour cell growth and malignant transformation. Hence, KLK6 may also serve as a therapeutic target for ovarian cancer. To further clarify KLK6's clinical utility and its role in ovarian cancer pathophysiology, we aimed to delineate the molecular processes underlying KLK6 overexpression in ovarian cancer.

We first conducted a validation study to confirm the prognostic significance of KLK6 in a large group of ovarian tumour tissues using a new immunoassay of higher sensitivity and specificity than previously reported (Diamandis *et al*, 2000). KLK6 protein was expressed at low levels in normal and benign ovarian tissues. Conversely, the KLK6 expression in ovarian tumour cytosols was significantly upregulated. This increase correlated with cancer stage (Figure 2B). Metastatic tumours to the ovary from primary gastrointestinal, endometrial, uterine, or breast cancers expressed low levels of KLK6 that were comparable with benign ovarian tissues. Hence, the up-regulation of KLK6 seems to be specific to ovarian cancer. Figure 2C indicates that most tumours with KLK6 up-regulation were of epithelial origin, and especially of serous and undifferentiated histotypes.

Univariate Cox regression analyses confirmed that KLK6 was significantly correlated with survival (Table 4). Kaplan–Meier survival curves confirmed that KLK6 was a significant and much stronger prognostic biomarker than CA125 for both PFS and OS (Figure 4). In multivariate Cox regression models adjusted for tumour grade, histotype, CA125 and patient age, the relationship between KLK6 expression and patient survival was no longer significant. Even though KLK6 may not be a strong independent prognostic indicator for ovarian cancer, it appears to be a surrogate marker for other clinicopathological variables such as tumour stage, grade, histotype and debulking success. Recent evidence indicates that multiparametric strategies that merge diagnostic and prognostic information provided by several individual biomarkers yield more informative and accurate medical predictions (Woolas *et al*, 1993, 1995; Zhang *et al*, 1999; Mor *et al*, 2005). Therefore, KLK6 could be used in conjunction with other kallikreins, as well as non-kallikrein biomarkers in future multiparametric prognostic tests for ovarian cancer.

To delineate the processes underlying the overexpression of KLK6 in ovarian cancer, we first determined whether KLK6 was under transcriptional or translational upregulation. We assessed *KLK6* mRNA expression in tumours with high or low KLK6 protein concentration and correlated these two parameters. Excellent concordance between KLK6 protein and mRNA expression was observed (Figure 5), rendering evidence for transcriptional regulation.

We evaluated the differential expression of the two most abundant transcripts of *KLK6*. Previous studies showed that ‘alternative transcript 1’ was expressed in both peripheral tissues (though the ovary was not examined) and the central nervous system, whereas the ‘classical transcript’ was only expressed in the central nervous system (Christophi *et al*, 2004; Pampalakis *et al*, 2004). We confirmed that the ‘classical transcript’ was highly expressed only in the brain, and weakly expressed or absent in all ovarian tissues. *KLK6* ‘alternative transcript 1’ was the dominant transcript in all samples examined (Figure 5). Therefore, differential expression of alternative transcripts cannot account for KLK6 upregulation in ovarian cancer.

Our genomic mutation screening experiments found no mutations except two novel exonic SNPs within a (GT)_4_ microsatellite in the 5′-UTR of ‘alternative transcript 1’ (+55 G/T, +60 G/T). The SNPs were not located in any putative promoter region or known steroid hormone response elements regulatory regions (Christophi *et al*, 2004). No consensual changes were observed within any sample types. Previous experiments have indicated a tendency for SNPs in the KLK locus to exhibit strong linkage disequilibrium (Cramer *et al*, 2003; Borgono and Diamandis, 2004). Indeed, these two SNPs were linked to each other.

Lastly, we evaluated the role of steroid hormones in the regulation of KLK6 in ovarian cancer. Sex steroid hormones have previously been implicated in the pathogenesis and growth regulation of ovarian cancers (Hoover *et al*, 1977; Langdon *et al*, 1990; Godwin *et al*, 1993; Chien *et al*, 1994). Hormonal regulation of KLKs in ovarian cancer has only been demonstrated in the ovarian cancer cell line, BG-1, where KLK9 was under the regulation of oestrogen and progesterone (Yousef *et al*, 2001). In this study, we found that steroid hormones play a minor role in the regulation of KLK6 in ovarian cancer. It remains to be elucidated why steroid hormones regulate KLK6 in breast cancer cell lines such as BT474 (Yousef *et al*, 1999), but not in receptor-positive ovarian cancer cell lines.

In conclusion, we confirmed that KLK6 is significantly upregulated in ovarian cancer and serves as an unfavourable prognostic biomarker. We also found that KLK6 is under transcriptional regulation. Nevertheless, the overexpression of KLK6 in ovarian cancer is probably not due to differential expression of alternative transcripts, mutations in the exons and proximal 5′-flanking region, or steroid hormone regulation.

Various other transcriptional regulation mechanisms remain to be tested for their roles in the upregulation of the KLKs in ovarian cancer. As subgroups of kallikreins are frequently coexpressed in tissues, transcription of *KLK* genes may be regulated by *cis*-acting locus control regions (Li *et al*, 1999). In fact, such a mechanism has been implicated in controlling the salivary gland-specific expression of the rat kallikrein gene family (Smith *et al*, 1992). Future studies should investigate whether a similar mechanism is at work in human ovaries. Furthermore, the promoter region of ‘alternative transcript 1’ has not been fully elucidated, and the actual transcription start site is not ascertained. Characterisation of the *KLK6* promoter would facilitate future efforts to study the regulation of KLK6, including studying the roles of transcription factors. Lastly, chromosome 19q13 is reportedly rearranged in numerous types of solid tumours (Whang-Peng *et al*, 1984; Tanaka *et al*, 1989; Bello and Rey, 1990; Pejovic *et al*, 1991, 1992; Jenkins *et al*, 1993). Potential gene amplification of KLK6 in ovarian cancer has also been suggested (Ni *et al*, 2004). Therefore, the role of chromosomal rearrangements of the *KLK* locus in cancer should be examined in future investigations.

## Figures and Tables

**Table 1:** Primer sequences used for PCR amplification and DNA sequencing

**Amplicon**	**Sequence (5′ → 3′)**	**PCR product length (bp)**	**Annealing temp (^o^C)**
	F: ATCTGGCACCACACCTTCTA		
*β*-Actin	R: CGTCATACTCCTGCTTGCTG	835	62
			
Four *KLK*6	F: GAAGCTGATGGTGGTGCTGAGTCTG		
Transcripts	R: AGACAGCAGATGGTGATTTCCCTGAC	454	65
			
Classical	F: AGGCGGACAAAACCCGATTGTTC		
Transcript	R: CTGCAGCAATCAGACTCAGCACCAC	285	61
			
Alternative	F: AGAAGCATCTGGGGACAGAACCAG		
Transcript 1	R: CTGCAGCAATCAGACTCAGCACCAC	290	61
			
*KLK*6	F: GTGCTGGGGGTGCAGGGAGG		
5′-Flanking	R: CCCCCACGCTCTGCTCTTGG	503	67
			
	F: CTCCCTTCCCTGGAGGCCTG		
Exon 2A+2	R: GGGAGTTTTCCTCGGAGCCTG	476	66
			
	F: GGAGAGGATGCAGAGGGAGCAGACA		
Exon 3	R: TCCCCAATACCAGCCTCTTCTCC	210	65
			
	F: ACTGGAGTTCATGTTGAGGGGGATG		
Exon 4	R: CCTATGTCACCTCCTGCCTGACATC	614	66
			
	F: CCCAGCTGGGGAAGACTGTGGG		
Exon 5	R: TGGCCCCTCACCAATTTTCCC	457	67
			
	F: GAGGGGTGCCAATAGAAAAGAGG		
Exon 6	R: GTGACTTCGTCCTGTCCCTGGCTG	314	66
			
	F: ATCTGACTTTCTCCCTCTTTCCTGC		
Exon 7	R: GTGCCTTCCTGGAGGGGAGG	816	65

F=forward, R=reverse.

Thirty-five cycles per PCR except for the ‘Four KLK6 Transcripts’ (32 cycles). (PCR=polymerase chain reaction).

**Table 2:** Tissue KLK6 concentrations in four groups of patients

**KLK6 (ng mg^−1^)**	**Mean±s.e.^a^**	**Median**	**Range**	***P*-value^b^**
Normal (*N*=34)	0.31±0.13	0.05	0.01–4.11	0.1^c^
Benign (*N*=49)	0.82±0.36	0.09	0.01–16.0	<0.001^d^
Ovarian cancer (*N*=259)	5.89±0.55	2.83	0.01–64.96	<0.001^e^
Nonovarian cancer metastatic to ovary (*N*=43)	0.83±0.28	0.09	0.01–8.56	<0.001^f^

a s.e.: Standard error.

b Calculated by Mann—Whitney test.

c Between normal and benign groups.

d Between normal and ovarian cancer groups.

e Between benign and ovarian cancer groups.

f Between ovarian cancer and nonovarian cancer metastatic to ovary.

**Table 3:** Relationship between tissue KLK6 status and other variables in ovarian cancer patients

		**No. of patients (%)**		
**Variable**	**Patients**	**KLK6 negative^a^**	**KLK6 positive**	***P*-value**
*Stage*				
I	65	47 (72.3)	18 (27.7)	<0.001^b^
II	20	9 (45.0)	11 (55.0)	
III	143	62 (43.4)	81 (56.6)	
IV	18	4 (22.2)	14 (77.8)	
x^c^	13			
				
*Grade*				
G1	58	33 (56.9)	25 (43.1)	0.04^b^
G2	46	28 (60.9)	18 (39.1)	
G3	139	59 (42.4)	80 (57.6)	
*x*^c^	16			
				
*Histotype*				
Serous	110	39 (35.5)	71 (64.5)	<0.001^b^
Endometrioid	46	30 (65.2)	16 (34.8)	
Mucinous	20	14 (70.0)	6 (30.0)	
Clear cell	18	12 (66.7)	6 (33.3)	
Undifferentiated	33	10 (30.3)	23 (69.7)	
Other nonepithelial	24	20 (83.3)	4 (16.7)	
*x*^c^	8			
				
*Debulking success* d				
SD	102	38 (37.3)	64 (62.7)	0.002^e^
OD	140	80 (57.1)	60 (42.9)	
*x*^c^	17			
				
*Response to CTX* f				
NC/PD	18	8 (44.4)	10 (55.6)	0.81^b^
PR	41	19 (46.3)	22 (53.7)	
CR	180	91 (50.6)	89 (49.4)	
NE	20			
				

a Cutoff=2.83 *μ*g mg^−1^ (50th percentile).

b *χ*^2^ test.

c Status unknown.

d OD: optimal debulking (0–1 cm), SD: suboptimal debulking (>1 cm).

e Fisher's exact test.

f CTX=chemotherapy; NC=no change; PD=progressive disease; CR=complete response; PR=partial response; NE=not evaluated.

**Table 4:** Univariate and multivariate analysis of tissue KLK6 with regard to PFS and OS in ovarian cancer

	**Progression-free survival**			**Overall survival**		
**Variable**	**HR^a^**	**95% CI^b^**	***P*-value**	**HR^a^**	**95% CI^b^**	***P*-value**
*Univariate analysis*						
KLK6						
Negative	1.00			1.00		
Positive	1.56	1.12–2.17	0.008	1.46	1.01–2.11	0.045
Continuous logarithmic variable	1.45	1.19–1.75	<0.001	1.37	1.11–1.71	0.004
CA125						
Negative	1.00			1.00		
Positive	1.13	0.82–1.57	0.45	1.08	0.75–1.55	0.68
Continuous logarithmic variable	1.15	0.96–1.38	0.11	1.05	0.85–1.27	0.66
Histological type^c^	1.95	1.40–2.72	<0.001	1.61	1.12–2.31	0.01
Stage (ordinal)	2.62	2.08–3.31	<0.001	2.52	1.92–3.29	<0.001
Grading (ordinal)	1.90	1.50–2.41	<0.001	1.58	1.09–2.28	0.014
Age (ordinal)	1.013	1.00–1.026	0.046	1.02	1.01–1.03	0.006
						
*Multivariate analysis*						
KLK6						
Negative	1.00			1.00		
Positive	1.04	0.73–1.49	0.83	1.04	0.69–1.56	0.84
Continuous logarithmic variable	1.15	0.88–1.51	0.28	1.24	0.92–1.68	0.16
CA125						
Negative	1.00			1.00		
Positive	0.94	0.66–1.34	0.74	0.92	0.62–1.37	0.68
Continuous logarithmic variable	0.87	0.66–1.15	0.34	0.75	0.54–1.03	0.08
Stage (ordinal)	2.29	1.75–2.99	<0.001	2.28	1.63–3.05	<0.001
Histological type^c^	1.15	0.81–1.65	0.43	0.99	0.66–1.47	0.96
Grading (ordinal)	1.27	0.97–1.68	0.078	1.31	0.94–1.80	0.11
Age (ordinal)	0.99	0.98–1.01	0.86	1.01	0.99–1.027	0.26

a Hazard ratio (HR) estimated from Cox proportional hazard regression model.

b Confidence interval of the estimated HR.

c Serous *vs* others.
